# The Emerging Role of Galectins and *O*-GlcNAc Homeostasis in Processes of Cellular Differentiation

**DOI:** 10.3390/cells9081792

**Published:** 2020-07-28

**Authors:** Rada Tazhitdinova, Alexander V. Timoshenko

**Affiliations:** Department of Biology, The University of Western Ontario, London, ON N6A 5B7, Canada; rtazhitd@uwo.ca

**Keywords:** galectins, *O*-GlcNAc, cellular differentiation, unconventional secretion, stem cells, signaling

## Abstract

Galectins are a family of soluble β-galactoside-binding proteins with diverse glycan-dependent and glycan-independent functions outside and inside the cell. Human cells express twelve out of sixteen recognized mammalian galectin genes and their expression profiles are very different between cell types and tissues. In this review, we summarize the current knowledge on the changes in the expression of individual galectins at mRNA and protein levels in different types of differentiating cells and the effects of recombinant galectins on cellular differentiation. A new model of galectin regulation is proposed considering the change in *O*-GlcNAc homeostasis between progenitor/stem cells and mature differentiated cells. The recognition of galectins as regulatory factors controlling cell differentiation and self-renewal is essential for developmental and cancer biology to develop innovative strategies for prevention and targeted treatment of proliferative diseases, tissue regeneration, and stem-cell therapy.

## 1. Introduction

Galectins are a family of soluble β-galactoside-binding proteins with diverse glycan-dependent and glycan-independent functions outside and inside the animal cell [1,2]. Galectins are identified by numbers (from -1 to -16) and 12 of them have homologues in human cells [3,4,5,6]. As such, galectins create a complex network of soluble proteins localized both outside cells and in different subcellular compartments (cytosol, mitochondria, nucleus). Many galectins are detected in human plasma (Supplementary Table S1) and usually bind and/or crosslink β-galactoside-containing glycans on the cell surface, thereby inducing transmembrane signaling for a variety of specific cellular responses essential for cell growth and death [7,8,9,10,11]. The most common galectin-binding ligand is Galβ1-4GlcNAc (*N*-acetyllactosamine), which is an epitope of more complex natural glycans with much stronger affinity to galectins. Moreover, extracellular galectins can interact with certain cellular receptors in a glycan-independent manner as demonstrated, for instance, by binding of galectin-1 to pre-B cell receptor [12] and galectin-9 to a pattern-recognition receptor dectin 1 [13]. Inside the cell, galectins function mostly through a glycan-independent mode, binding allosterically to regulatory proteins that control pre-mRNA splicing, signal transduction, gene expression, and apoptosis [10,14]. However, intracellular glycan-dependent interactions of galectins can also occur, targeting luminal glycoproteins of damaged phagosomes and lysosomes in an association with autophagy processes [15,16,17,18,19]. The multifunctional nature of galectins makes them critical players in many biological processes such as embryogenesis, angiogenesis, neurogenesis, immunity, and tissue regeneration, which are not always clearly defined.

Two important advances in galectin biology provide a framework for better understanding of the biological significance of galectins in the context of developmental and cell biology. First, galectin expression profiles vary significantly between different cell types and tissues [4,5,20,21]. Indeed, while some galectins (e.g., galectins 1, 3, and 8) are commonly expressed by many types of tissues (Supplementary Figure S1), others are exclusive to specific cells or tissues, e.g., galectin-12 in myeloid cells and adipocytes [22] or galectins-13, -14, and -16 in the placenta [4]. Second, lineage-specific remodeling of galectin networks during cell differentiation is associated with both upregulation and downregulation of individual galectins in different model systems including hematopoietic cells, adipocytes, muscle cells, and more [22,23,24,25,26]. Thus, the tissue-specific signature of galectin expression suggests the galectin network may act as through mechanisms controlling cellular differentiation and stem cell self-renewal that requires a systematic review of relevant participation or involvement of galectins. 

Emerging evidence suggests that one of the hallmarks of differentiated cells in comparison with progenitor cells is a decrease in glycosylation of intracellular proteins with *N*-acetyl-d-glucosamine, termed *O*-GlcNAcylation [27,28,29,30,31]. *O*-GlcNAcylation is known to change the functional activities of proteins and govern the trafficking and localization of proteins in cells. Recently, we have proposed that the regulatory functions of galectins toward cell self-renewal and differentiation depend on the localization of galectins in cells which may be driven by *O*-GlcNAc signaling mechanisms [31,32]. In particular, we propose that high levels of *O*-GlcNAc promote the intracellular accumulation of galectins in stem cells while low levels of *O*-GlcNAc promote galectin secretion and cell differentiation.

This review aims to explore the available evidence on the potential involvements of individual galectins in processes of cellular differentiation and to explore formal links to relevant *O*-GlcNAc cellular homeostasis.

## 2. Galectin-1

Galectin-1 is one of the most commonly expressed galectins, found throughout the human body in different cell types and tissues, including stem cells. The Human Protein Atlas (HPA) classifies the gene to a category of “low tissue specificity” based on transcriptomics analysis (available from www.proteinatlas.org, Supplementary Figure S1A). Galectin-1 usually has a higher concentration in circulation in comparison with other galectins (Supplementary Table S1) reaching a median level of 328.8 ng/mL (range 56.6–11670.2 ng/mL) in the sera of healthy individuals [33]. The involvement of endogenous and recombinant galectin-1 in regulating cell differentiation was reported for many human cell lineages including muscle cells, trophoblasts, immune cells, astrocytes, osteoblasts, and others (Table 1 and Table 2). Galectin-1 was also reported to induce proliferation of stem cells, including mouse embryonic stem cells and adult neural stem cells [34,35]. Many aspects of galectin-1 functions including cell differentiation were covered by Camby and coauthors [36] and we present here the current update of this knowledge focusing more on human cell lines.

### 2.1. Muscle Cells

Galectin-1 is expressed in different types of muscle tissues [98] and essential for muscle functioning due to its association with sarcomeric actin on I bands [99] and overall high binding affinity to cytoplasmic actin [100]. Many processes of muscle development are associated with elevated levels of galectin-1 leading to myoblast fusion, terminal differentiation, and muscle regeneration [24,76,77,101]. Interestingly, galectin-1 is mostly localized in the cytoplasm of myoblasts whereas it is released in extracellular vesicles from terminally differentiated multinucleate myotubes [56,57]. Primary mouse myoblasts and C2C12 cells (a mouse myogenic cell line), which were treated with galectin-1-enriched cell culture medium or recombinant galectin-1, demonstrated signs of terminally differentiated muscle cells such as elevated levels of desmin and multinucleate morphology [76,77]. The stimulatory role of extracellular galectin-1 in myocytic differentiation is further confirmed in experiments with fetal mesenchymal stem cells of both human and bovine origin. Indeed, direct treatments of these stem cells with galectin-1 stimulate the expression of myogenic regulatory factors (e.g., Myf5, Pax7, MyoD, and myogenin) and muscle differentiation [24,75]. Similarly, transfection of rat mesenchymal stromal cells from bone marrow with galectin-1 induced their differentiation into skeletal muscle cells [102]. 

### 2.2. Trophoblasts

Many studies suggest that normal placental development [103,104] and the severity of preeclampsia [105] depends on galectin-1. Trophoblasts are specialized cells of the placenta which proliferate and differentiate into different cell subsets such as syncytiotrophoblasts, villous cytotrophoblasts, and distinct extravillous cytotrophoblasts with distinct functions [106]. These cell lineages express different levels of galectin-1 overall demonstrating increasing levels of galectin-1 in extravillous cytotrophoblasts essential for invasion and migration activities towards the decidua [107,108,109]. Upregulation of galectin-1 in human and animal trophoblastic cells can be induced by a variety of cell differentiation stimuli including sodium butyrate [110] and 8-Br-cAMP [111]. Studies with human choriocarcinoma cell line BeWo revealed that extracellular galectin-1 inhibited trophoblast cell proliferation and induced syncytium formation, which was associated with modulating activity of MAPK signaling pathway, the receptor tyrosine kinases, Janus kinase 2 (JAK2), and vascular endothelial growth factor receptor 3 [82]. In support of these findings, knockdown of galectin-1 gene in BeWo cells significantly reduced the rate of syncytium formation [112]. Recent studies also justified that increasing levels of galectin-1 protein and mRNA are associated with mouse trophoblast stem cells (TSC) differentiation into three different lineages of trophoblast giant cells, syncytiotrophoblasts, and spongiotrophoblasts [68]. This increase in galectin-1 protein and mRNA expression was observed during the early stages of TSC differentiation and reached peak levels on days 4 and 5 [69]. Recombinant galectin-1 promoted differentiation of TSC as seen by the down-regulation of TSC marker genes such as *Esrrb* and *Eomes* [68], which was consistent with the stimulatory and inhibitory effects of galectin-1 overexpression and knockdown, respectively [69]. 

### 2.3. Blood Cells

Hematopoietic stem cells give rise to two important lineages of blood cells including myeloid and lymphoid cells. The involvement of galectin-1 in mechanisms of hematopoietic stem cell differentiation has been reported in several cell models highlighting the important role of this factor in myelopoiesis, lymphopoiesis, and erythropoiesis. 

The human HL-60 cell line is often used as a model of myeloid cell differentiation producing neutrophil-, eosinophil-, or macrophage-like cells depending on differentiation stimuli. There is evidence that galectin-1 is readily expressed in HL-60 cells, however no changes were reported at the mRNA level in differentiated cells [23,26]. Surprisingly, we noticed a 4-fold increase of intracellular galectin-1 protein expression in neutrophil-like cells, differentiated from HL-60 cells by 1.3% dimethyl sulfoxide (DMSO) [26]. This finding highlights a discrepancy between mRNA and protein levels for galectin-1, which was also noticed in an animal model of hypometabolism [113] suggesting additional regulatory mechanisms of galectin transcription and translation, e.g., miRNA-dependent galectin mRNA stability [114,115]. As galectin-1 is also found at high concentration in circulation, its abundance might be essential to maintain the functional activity of neutrophils by inducing plasma membrane NADPH activity and cell degranulation as we demonstrated in previous studies [7,115,116,117]. However, these aspects of functional activity of extracellular galectin-1 can vary in a biphasic manner depending on concentration as demonstrated for granulocytic differentiation [74] and respiratory burst of neutrophils [118]. The reason for these biological variations is not clear, although the role of glycan-dependent and glycan-independent interactions was proposed [74]. Studies with other stem-like leukemia cells (U937, U937T, and NB4) also confirmed a stimulatory role of galectin-1 in a model of hypoxia-induced granulocytic differentiation [46]. In this model, however, the role of galectin-1 was essential but not sufficient for the myeloid differentiation regardless of whether the cells were treated with the recombinant galectin-1 (10 µg/mL) or the cells were under hypoxic conditions inducing the expression of galectin-1. In comparison, the differentiation of human peripheral monocyte cells into tolerogenic dendritic cells was readily induced by galectin-1 used at a concentration of 1 µg/mL [81]. Although galectin-1 seems to be one of the regulatory factors required for the myeloid differentiation pathway, its expression at protein and gene levels was not changed in classically (M1) and alternatively (M2a and M2c) activated macrophages from human monocytes [48]. 

Galectin-1 is also involved in the lymphopoietic processes leading to generation of mature T cells and B cells. For instance, CD4^+^CD25^+^ regulatory T cells are characterized by increasing expression of galectin-1 in a model of T-cell receptor (TCR) activation [119]. The overexpression of galectin-1 is important to maintain the functions of this T cell population as neutralizing galectin-1 antibody blocks in vitro their suppressive activity related to regulating autoimmune responses. In more general context, TCR receptor presence seems to be an essential factor for galectin-1 to modulate overall thymocyte development through transient activation of the ERK pathway [120]. In comparison, CD69 was found to be a key galectin-1-binding receptor in CD4^+^ T cells responsible for inhibiting Th17 cell differentiation by recombinant galectin-1 (10 µg/mL) since CD69-deficient cells were unresponsive [80]. 

Several lines of evidence suggest that galectin-1 is a positive regulator or at least an associated factor in plasma cell differentiation. Indeed, treatment of the BL36 B lymphoma cell line with 5-azacytidine, which is a DNA methyltransferase inhibitor cleaning up the methylation of CpG clusters in the promoter region of galectin-1 gene [121], induced both galectin-1 protein expression and plasmatic differentiation of the cells as revealed by overexpressing a plasma cell marker CD138 [64]. Comprehensive studies with mature B cells further established that galectin-1 as well as galectin-8 are positive regulators while galectin-3 is a negative regulator of terminal B cell differentiation into plasma cells [122]. The expression of these galectins at protein levels in mature B cells was accordingly increased or decreased in response to lipopolysaccharide (LPS)-induced plasma cell differentiation [65,66]. Interestingly, a discrepancy between changes at protein and mRNA levels (mRNA decrease versus protein increase) was noticed for galectin-8 suggesting that the stability of this protein increased during differentiation. As such, both galectin-1 and -8 stimulated differentiation of B cells into immunoglobulin-producing cells through an extracellular mechanism, since recombinant galectins had a stronger binding affinity to B cells rather than to plasma cells and galectin-binding glycans inhibited the plasma cell differentiation. Thus, galectin-1 and galectin-8 work in concert with each other in this context, while the role of galectin-3 seems to be different facilitating the formation of memory B cell rather than plasma cells [123]. 

The involvement of galectin-1 in mechanisms of erythropoiesis was demonstrated in a model of human leukemia cell line K562, which constitutively expresses this lectin in cytoplasm. The erythroid differentiation induced by erythropoietin or aphidicolin resulted in a drastic decrease of galectin-1 in cytosol and a strong increase on the cell surface (externalization or secretion) [45]. The pool of secreted galectin-1 is available for autocrine regulation of cellular differentiation and further evidence with recombinant and knockdown galectin-1 is warranted in this context. As galectin-1 is a possible target of specific posttranslational glycosylation with *O*-linked *N*-acetyl-d-glucosamine called *O*-GlcNAcylation [124], the externalization of galectin-1 could be explained in the framework concept about *O*-GlcNAc-mediated redistribution of galectin-1 between intra- and extra-cellular space [32]. As such, progenitor cells keep a high level of global *O*-GlcNAcylation in comparison to differentiated cells [31], which might prevent the secretion of soluble proteins like galectins. Similar mechanisms may work in the case of mouse 3T3-L1 pre-adipocyte to adipocyte differentiation, which is readily accompanied by an increase in galectin-1 secretion [37].

### 2.4. Glial Cells

Glial cells are the most abundant cell types in the central nervous system, which include oligodendrocytes, astrocytes, Schwann cells, ependymal cells, microglia, and satellite cells. The variable role of galectin-1 was demonstrated in regulating astrocyte and oligodendrocyte differentiation. Recombinant galectin-1 at a concentration of 10 µM readily induced astrocyte differentiation, which was accompanied by a characteristic cell morphology change from an immature polygonal to a matured stellate shape, inhibiting cell proliferation, and increasing expression of biomarkers of differentiated cells such as glial fibrillary acidic protein (GFAP) and brain-derived neurotrophic factor [71]. It is interesting that galectin-1-induced differentiation of astrocytes was resistant to treatments with a protein kinase A inhibitor KT5720 and a tyrosine kinase inhibitor genistein while an inhibitor of protein tyrosine phosphatase orthovanadate almost completely inhibited the stellation of astrocytes indicating a key role of global phosphorylation of relevant intracellular proteins in this process. This aspect of regulation may provide an advantage condition for competitive *O*-GlcNAcylation of intracellular proteins that can support the undifferentiated state of different cell types [31]. 

However, recombinant galectin-1 at concentrations of 5 µg/mL and 10 µg/mL inhibited primary oligodendrocyte differentiation as the expression of myelin basic protein (MBP) isoforms (markers of mature oligodendrocytes) was significantly decreased [58]. Recent study demonstrated that the concentration of galectin-1 can be crucial in the differentiation patterns of glial cells as this galectin can function differently in monomeric (~50 µg/mL) or dimeric forms (~125 µg/mL). Indeed, only treatment of primary glial cells with 125 µg/mL of recombinant galectin-1 increased the expression of specific markers of oligodendroglial differentiation (NG2 and MBP) while no changes in the expression of GFAP (marker of astroglial differentiation) was detected [78]. Taking into account the importance of the cross-talk between astrocytes and oligodendrocytes in the central nervous system [125], these findings suggest that galectin-1 could be a modulatory factor of glial development. This regulation can be very complex considering that galectin-1 also facilitates the generation of neural crest cells, which are multipotent cells from the dorsal region of the neural tube in embryos giving rise to various cells including neurons and glial cells [126].

### 2.5. Bone and Cartilage Cells

Osteogenic and chondrogenic cell lineages are originated from human mesenchymal stem cells (MSC), which have been originally isolated from bone marrow [127]. Galectin-1 was reported as a stimulatory factor in the context of both types of MSC differentiation. For instance, recombinant galectin-1 used at concentrations of 10 and 1000 ng/mL inhibited proliferation of human bone marrow stromal cells whereas the activity of alkaline phosphatase, a marker of early osteoblast differentiation, was increased in conjunction with decreasing secretion of osteocalcin, a marker of later osteoblast differentiation [79]. The effect of recombinant galectin-1 on chondrogenic differentiation of bone marrow-derived human MSC was clearly dose-dependent/biphasic and observed only at relatively low concentrations of 0.4–2.0 µg/mL whereas higher concentrations (2.0–10.0 µg/mL) inhibited chondrogenesis [72]. Biphasic activity of galectin-1 is also well-known in regulating processes of cell proliferation and apoptosis [128] that require careful consideration if applied for cellular and bone tissue therapy.

## 3. Galectin-2

Galectin-2 is a group-enriched galectin as per the HPA classification, which is expressed at protein and mRNA levels in the gastrointestinal tract, bone marrow and immune system, liver and gallbladder, pancreas, blood, and the kidney (Figure S1B). This galectin is detected in the serum of healthy donors at concentrations of 2.2–914.8 ng/mL (median 14.1 ng/mL) and the serum levels are moderately elevated in patients with breast and colorectal carcinoma [33]. The participation of galectin-2 in cell differentiation was only reported for CD14-expressing human monocytes and macrophages (Table 2). Treatment of these cells with recombinant galectin-2 (10 µg/mL) stimulated their polarization to a pro-inflammatory M1-like phenotype and prevented pro-arteriogenic M2 differentiation [83]. In terms of *O*-GlcNAc biochemistry and cellular differentiation, M1 phenotype is associated with lower levels of *O*-GlcNAcylation than M2 phenotype [129]. Thus, galectin-2 favors differentiation of macrophages with lower *O*-GlcNAc homeostatic levels that could be a cell type-specific effect. Indeed, the role of galectin-2 might be more complex as its circulating levels were reported to increase moderately in patients with breast cancer (median 1.2-fold increase) and colorectal cancer (median 1.9-fold increase) [33] whereas galectin-2 expression was decreased in the placental tissue of patients with preeclampsia and spontaneous abortions [130,131].

## 4. Galectin-3

Galectin-3 functions in regulating cell differentiation have been widely examined since the late 1990s. It is one of the most commonly expressed galectins, with evidence of both mRNA and protein expression found throughout all tissues (low tissue specificity protein as per HPA classification) of the human body (Supplementary Figure S1C). Galectin-3 is localized in the cytoplasm and nucleus and can also be secreted from cells by non-classical mechanisms reaching the median level in plasma at a baseline of 10.8 (9.0–13.0) ng/mL [132]. A level of galectin-3 in blood plasma higher than 17.8 ng/mL is considered a risk factor in patients with acute heart failure requiring more intensive therapy to avoid rehospitalization [133]. Although most commonly known for its role in bone cell differentiation [134], it is also involved in the differentiation of immune cells, adipocytes, and oligodendrocytes (Table 1 and Table 2). There is also evidence that galectin-3 contributes to regulating cell stemness-related signaling pathways and maintaining cancer stem cells [135,136,137]. 

### 4.1. Bone and Cartilage Cells

Galectin-3 is highly expressed in bone and cartilage cells, including osteoclasts, osteoblasts, osteocytes, and chondrocytes [62,138,139]. A recent review by Iacobini et al. [134] provides a comprehensive summary of galectin-3 roles in the differentiation of osteogenic and chondrogenic cell lineages and its relationship to inflammatory bone and joint diseases. Many evidences indicate that galectin-3 is a promoting factor and is required for various aspects of bone cell differentiation and bone remodeling, which rely on bone-specific RUNX2 and RANKL signaling pathways. Galectin-3 works together with intracellular regulatory molecules to promote osteogenic differentiation as galectin-3, TRIM16 (tripartite motif protein 16), and ULK1 (Unc-51 like autophagy activating kinase 1) knockdown in human bone marrow-derived mesenchymal stem cells decreased the expression of pro-osteogenic markers such as RUNX2, osterix, osteocalcin, and alkaline phosphatase [140]. However, some studies report inhibitory effects of extracellular galectin-3 on osteoblast differentiation [61,85] and osteoclast differentiation [63,86]. These observations may reflect different functions of galectin-3 depending on its intracellular or extracellular localization, which is a well-known phenomenon [141,142]. Remarkably, osteoblast differentiation was associated with or induced by increasing global *O*-GlcNAc levels in a model of mouse preosteoblastic MC3T3-E1 cells [143,144], which might favor the intracellular accumulation of galectins [32]. This mechanism may be different in the case of osteoclasts because osteoclast differentiation of RAW264 monocytic cells was unaffected by treatment with *O*-GlcNAcase inhibitors [144].

### 4.2. Immune Cells

The expression of intracellular galectin-3 was increased in HL-60 cells undergoing neutrophilic differentiation induced by high dose DMSO [26]. Intracellular signaling related to oxidative stress, DNA methylation, and PP1/JNK/Sp1 signaling pathway might be responsible at least in part for the upregulation of galectin-3 [26]. The expression of galectin-3 was gradually increased in maturing myeloid cells isolated from human bone marrow and more galectin-3 was detected on the cell surface, which might represent secreted galectin-3 [55]. The myeloid progenitor cells from the bone of galectin-3-knockout mice showed a limited capacity to differentiate into mature myeloid and monocyte cell populations [145], while mesenchymal stromal cells with galectin-3 knockdown were able to differentiate into adipocytes, osteocytes, and chondrocytes [146]. In comparison, galectin-3-deficient mouse hepatic progenitor cells were more prone than primary cells to differentiate into biliary cells and mature hepatocytes demonstrating a lineage-dependent role of galectin-3 [147]. Recombinant galectin-3 binding to CD34^+^ progenitor myeloid cells was very efficient and resulted in a dose-dependent stimulation (<4 µg/mL) or inhibition (>4 µg/mL) of the cell proliferation, which represented biphasic activity of this galectin [55]. The increase in the expression of galectin-3 coincided with decreasing levels of *O*-GlcNAc in high DMSO-treated HL-60 cells leading to neutrophilic differentiation [23,31]. Lowering *O*-GlcNAc with pharmacological inhibitors also induced differentiation of acute myeloid leukemia OCI-AML3 and HL-60 cells [148]. Thus, the inverse relationship between secretion of galectin-3 and the global levels of *O*-GlcNAc seems to be a novel feature of differentiating myeloid cells.

Similar changes in galectin-3 expression as in neutrophilic differentiation were observed in the case of differentiation and activation of macrophages. A comprehensive study by Liu and coauthors [149] was the first that described important aspects of spontaneous in vitro differentiation of human peripheral blood monocytes into macrophages in association with a drastic increase of total galectin-3 in cells and on the cell surface. Macrophages can be polarized into two different subtypes of classically activated and pro-inflammatory M1 macrophages and alternatively activated and anti-inflammatory M2 macrophages [150]. It was demonstrated that, independently of the type of activation, the levels of galectin-3 were increased in differentiated macrophages, namely by 6.8-fold in M1 phenotype and by 16.7-fold and 21.0-fold in cases of M2a and M2c phenotypes, respectively [48]. The changes of the galectin-3 mRNA levels were less pronounced, showing a ~3-fold elevated expression in alternatively activated macrophages and no change in classically activated macrophages in comparison with monocytes [48]. These observations again highlight a role of post-translational regulation of galectins in cells. A role of *O*-GlcNAc-mediated mechanisms might be important in this context because the surface/extracellular galectin-3 levels were lower in monocytes than in both types of macrophages, although being slightly higher in M2 phenotype [48]. Studies with human leukemia cell cultures (HL-60 and THP-1) differentiating into macrophages by phorbol-12-myristate-13-acetate (PMA) reported the same trends of upregulating galectin-3 at protein and mRNA levels as with native blood monocyte cells [23,51,52,54]. THP-1 cells were also differentiated into a M1/M2 mixed macrophage-like phenotype with increasing levels of galectin-3 in response to condition medium from colorectal carcinoma cell lines (HT29, LS180, SW948, SW620), suggesting the relevance of these mechanisms to the functioning of tumor-associated macrophages [49]. Monocytes can also differentiate into fibrocytes, which are required for healing wounds and fibrotic lesions, and galectin-3 was found to potentiate this process competing with galectin-3 binding protein [73]. Similarly, differentiation of CD34^+^ mononuclear cells into endothelial progenitor cells was also positively associated with upregulation of galectin-3 mRNA [42,43]. It should be noted that in a different but relevant model, treatment of pulmonary artery endothelial cells with endogenous galectin-3 drove transdifferentiation to vascular smooth muscle cells, through Jagged1/Notch1 pathways, all confirming a differentiation-promoting role of galectin-3 [87]. Notably, however, that differentiation of human monocytes into macrophages versus dendritic cells [151] might utilize galectin-3 differently as this lectin was reported to impair GM-CSF–induced differentiation and reduced antigen-presenting capacity of dendritic cell in patients with leprosy [84]. 

Galectin-3 seems to play an opposite role in plasma cell versus memory B cell differentiation as its expression in germinal center B cells and plasma cells was much lower than in naïve B cells and memory B cells from human tonsils [152]. As such, galectin-3 favored a memory B cell phenotype rather than plasma cell as demonstrated in splenocytes treated with specific differentiation stimuli (IL-2, IL-4, CD40 cross-linking) or genetically modified using an antisense strategy to suppress galectin-3 expression [123]. Accordingly, the level of galectin-3 mRNA was decreasing in splenic B cells treated with LPS mimicking the T cell-independent plasma cell differentiation [66]. An inhibitory role of galectin-3 was demonstrated in a model of galectin-3 knockout mouse, where B lymphocytes of the peritoneal cavity favored differentiating into plasma cells [153]. More specifically, a significant increase of IgA-producing cells in the peritoneal cavity was observed in comparison to galectin-3-expressing mice during chronic schistosomiasis as well as in response to interleukin-5 and transforming growth factor-β [154]. Although the mechanisms of galectin-3-dependent inhibition of B cell differentiation are obscure, a recent study revealed that Notch signaling pathways might play an important regulatory role [155]. 

### 4.3. Adipocytes

Galectin-3 is well-known to stimulate differentiation and maturation of adipocytes and is associated with obesity in humans and mouse models [40,156,157]. The expression of galectin-3 increased in a time-dependent manner in both human subcutaneous preadipocytes and mouse 3T3-L1 cells undergoing differentiation into mature adipocytes in cell culture [38,40]. Stable silencing of galectin-3 in 3T3-L1 cells resulted in a significant inhibition of adipocyte differentiation and lipid accumulation in comparison with control 3T3-L1 cells and had a low expression of biomarkers and transcription factors related to lipid metabolism such as PPARγ, C/EBPα, C/EBPβ, and FABP4 [40]. The potential effects of free fatty acids such as palmitate, oleate and linoleate on galectin-3 expression in adipocytes were also examined. 3T3-L1 cells differentiated with a high concentration of linoleate (200 μM) increased the levels of both intracellular and secreted galectin-3, and linoleate had the largest effect on galectin-3 compared to palmitate and oleate [38]. It is pertinent to note that inhibition of galectin-3 expression using biochemical compounds, e.g., barberine, suppressed the differentiation of mouse primary preadipocytes [158]. These in vitro findings provide a support for the clinical testing of galectin-3 inhibitors for the treatment of obesity or related diseases.

### 4.4. Oligodendrocytes

Galectin-3 presented abundantly in astrocytes, microglia, and oligodendroglial cell line N20.1 and its expression was significantly upregulated during differentiation of primary oligodendrocyte cells, which had a low basal level of galectin-3 [58]. Differentiation of oligodendroglial progenitor cells was efficiently induced by both recombinant galectin-3 and by galectin-3-contained condition medium from microglial cell culture confirmed by an increase in the number of cells positively stained for MBP, a marker of mature oligodendrocytes [58]. Molecular mechanisms of galectin-3-induced oligodendrocyte differentiation are complex and associated with actin cytoskeleton assembly and a decrease in Erk1/2 activation at early stages followed by the activation of Akt signaling in mature cells [159,160]. 

## 5. Galectin-4

Galectin-4 is a group-enriched galectin, with high mRNA and protein expression in the gastrointestinal tract, as well as some protein expression in the liver, gallbladder, and the lymphoid tissues (Figure S1D). In the gut epithelium, galectin-4 is specifically localized in the absorptive epithelial cells of the middle part of villi [21]. This galectin has intracellular localization, however can be secreted and detected in serum at mean concentrations of 79 pg/mL [161] and 238 pg/mL [162], and a median concentration of 3.9 ng/mL which was increased up to 25-fold in patients with breast and colorectal cancer [33]. Galectin-4 may play a role in regulating differentiation in trophoblast and oligodendrocyte cells, having a similar inhibitory effect in both (Table 1). It is also considered a marker for several forms of cancer, including colorectal, hepatocellular and gastroenteropancreatic neuroendocrine tumors [161,163,164]. As poor differentiation is a hallmark of cancer cells, these studies may suggest that galectin-4 might support mechanisms preventing cell differentiation events. However, this notion is most likely not general in the context of different tissues and cells as there is evidence that galectin-4 may induce macrophage and enterocyte differentiation.

### 5.1. Enterocytes and Macrophages

Differentiation of human colorectal carcinoma cell lines LS174T and HT-29 into enterocyte-like lineage is accompanied by increasing galectin-4 mRNA expression [31,44]. This upregulation could be due to the repression of heparin/heparan sulfate interacting protein/ribosomal protein L29 (HIP/RPL29), which is upregulated in colon cancer tissue [44]. Recombinant galectin-4 at a high concentration of 50 µg/mL induced growth inhibition and a more differentiated cell phenotype in five colon carcinoma cell lines including LS 180, Vaco 432, Colo 205, CX 1, and HCT 116 [165]. These observations are consistent with a tumor suppressor role of galectin-4 in colorectal carcinoma considering a decrease of mRNA expression in early stages of tumor formation [163] and an increase of HT-29 cell proliferation due to a knockdown of galectin-4 that interferes with Wnt signaling in colorectal cancer cells [166]. Upregulation of galectin-4 during enterocytic differentiation is strongly correlated with decreasing global *O*-GlcNAc in cells [31], which can indeed deregulate Wnt signaling [167]. 

A stimulatory role of galectin-4 was demonstrated in the case of macrophage-like differentiation of human monocytes. Galectin-4 at a high concentration of 10 µg/mL activated a CD14-mediated cascade of changes in monocytes coupled with activation of MAPK signaling and phosphorylation of p38, JNK, and ERK and the expression of specific markers of macrophage phenotype [88].

### 5.2. Oligodendrocytes and Trophoblasts

There is evidence that neuronal galectin-4 is a negative regulator of myelination through inhibition of oligodendrocyte differentiation. The expression of galectin-4 mRNA was relatively high in rat progenitor oligodendrocytes, cortical neurons, microglia, and astrocytes whereas barely detectable in mature oligodendrocytes [59]. Moreover, galectin-4 expression was down-regulated in developing rat brain with the onset of myelination. The reappearance of neuronal galectin-4 is suggested to disrupt the remyelination process and is found to be constantly present in multiple sclerosis lesions in humans [168]. Neuronal galectin-4 interfered with rat oligodendrocyte differentiation and might promote dedifferentiation and proliferation. Indeed, treatment of immature oligodendrocyte with recombinant galectin-4 (0.05–2 µM) resulted in dose-dependent changes showing morphologically less differentiated cells after 4 days and impaired differentiation as per the decreasing expression of MBP [59]. This effect was also reversible, as the cells were able to differentiate normally again once galectin-4 treatment was withdrawn.

The negative role of galectin-4 in trophoblast differentiation was reported in a rat model system of Rcho-1 trophoblast stem cells. The expression of galectin-4 mRNA was drastically reduced on day 1 and 5 post-differentiation of Rcho-1 cells while galectin-1, -3, -8, and -9 were unaffected [70]. However, galectin-4 may play a different role in differentiation of different types of placental cells because in rat placenta, weak staining for galectin-4 was observed in syncytiotrophoblasts while a relatively strong staining in trophoblast giant cells [70]. Autophagy was proposed as a mechanism regulating galectin-4 expression in trophoblasts. Indeed, autophagy inhibitors (3-methyladenine and bafilomycin A1) abolished down-regulation of galectin-4 and suppressed differentiation of Rcho-1 cells into invasive trophoblasts [169]. 

## 6. Galectin-7

Galectin-7 is a group-enriched galectin as per the HPA classification with protein expression present in squamous epithelial cells of the esophagus and oral mucosa, skin, female tissues, and tonsils (Figure S1E). This galectin has intracellular localization, however, can be secreted and detected in the serum of healthy individuals at a range of 19.5–341.8 pg/mL [170]. Galectin-7 is positively associated with differentiation of keratinocytes (Table 1).

Galectin-7 is a marker of stratified squamous epithelium in the gut and skin [21,171,172]. Galectin-7 mRNA is found at all stages of epidermal differentiation, including the proliferative basal and differentiated suprabasal compartments [173]. The levels of galectin-7 mRNA were up-regulated in confluent culture of primary human keratinocytes undergoing terminal differentiation as confirmed by concomitant upregulation of corresponding differentiation markers keratin 1 and keratin 10 mRNAs [47]. Interesting that the change in the expression of galectin-1 was opposite to galectin-7 and galectin-3 that highlighted different role of these galectins in keratinocyte differentiation. In a human aneuploid immortal keratinocyte cell line HaCaT, galectin-7 knockdown resulted in a reduced keratinocyte differentiation and increased proliferation, which was accompanied by a decrease in expression of keratin-1 and keratin-10 mRNAs [174]. In this model, galectin-7 was shown to utilize the JNK1-miR-230-p63 signaling pathway as all these molecules were involved in keratinocyte differentiation. A weaker immunostaining of galectin-7 was also noticed in poorly versus highly differentiated tissues from patients with esophageal squamous cell carcinoma as well as in normal versus cancer tissues [175]. This apparent inconsistency could reflect localization heterogeneity and functional implication of galectin-7 as it had primarily nuclear localization in normal cells, whereas it was detected ubiquitously in the cytoplasm, nuclei, and membranes of cancer cells.

As galectin-7 is predominantly expressed in squamous epithelial cells and keratinocytes in the epidermis, there has been limited focus thus far on its involvement in the differentiation of other cell types. One study reported that galectin-7 can block TGF-β-mediated effects, which can have a positive effect on Th1 cell differentiation [89]. Another study found that galectin-7 had no inhibitory effect on osteoclastogenesis, as opposed to galectin-3 which had a suppressive role [86]. 

## 7. Galectin-8

Galectin-8 has widespread mRNA and protein expression throughout all tissue types and cells of the body and is classified by the HPA as a low tissue specificity gene (Supplementary Figure S1F). Concentration of galectin-8 in human serum ranged from 0.1 to 166.3 ng/mL (median 6.3 ng/mL) and increased up to five-fold in patients with colorectal and breast cancer [33]. Galectin-8 is involved in the differentiation of several cell types including T cells, plasma cells, and bone cells while lacking detected involvement in myeloid cell differentiation (Table 1). Recombinant galectin-8 can also stimulate differentiation of several cell types (Table 2).

### 7.1. T Lymphocytes and Plasma Cells

Galectin-8 shares a similar and redundant role with galectin-1 in plasma cell differentiation, although these galectins may function through partially different pathways. In a model of LPS-induced plasma cell differentiation of splenic B cells, both treatment of cells with recombinant galectin-8 (0.5 and 1.0 µM) and ectopic expression of galectin-8 resulted in efficient generation of plasma cells [66]. It is interesting that galectin-8 is constitutively expressed in plasma cells and experiences an increase at the protein level in differentiating B cells, whereas the level of galectin-8 mRNA drops down during this process [66]. This suggests that the stability of galectin-8 might increase in differentiated cells, a phenomenon deserving attention with other galectins at organismal level. Indeed, we observed an enhanced stability of galectins in an animal model of hibernation [113]. Differentiated plasma cells secreted 2–3-fold more galectin-8 and galectin-1 in comparison with untreated B cells [66] that may result from an inhibitory influence of *O*-GlcNAc on secretory process in cells. The role of extracellular galectin-8 seems to be important in plasma cell differentiation as this process was blocked by specific carbohydrate inhibitors of galectin binding [66]. 

Recombinant galectin-8 was shown to promote differentiation of splenic CD4^+^ T cells into two specific subtypes of T cells (regulatory T cells and T-helper type 2 cells, T_H_2) through the activation of IL-2 and TGF-β signaling pathways coupled with phosphorylation of STAT5 and Smad3 [92]. These effects of galectin-8 were dose-dependent (concentration range of 0.1–1.5 µM) and glycan-binding-specific (inhibition by thiodigalactoside). In comparison, no effects on T_H_1 differentiation and reduced T_H_17 polarization were noticed in the presence of recombinant galectin-8 [92]. 

### 7.2. Bone Cells

Galectin-8 also plays an indirect role in the differentiation of osteoclasts. A study found that osteoblasts co-cultured with bone marrow cells and treated with galectin-8 (50 nM) had a 15-fold increase in osteoclast differentiation [91]. However, galectin-8 on its own had no effect on osteoclast differentiation, as bone marrow cells treated with galectin-8 in the absence of osteoblasts did not undergo differentiation [91]. In fact, osteoblasts serve as a source of an osteoclastogenic factor RANKL, expression of which increased by at least six-fold in the presence of galectin-8. Moreover, galectin-8 inhibits osteoblast differentiation and stimulates bone resorption in a mouse model as a result of impaired osteoclast/osteoblast balance [90]. 

### 7.3. Myeloid Cells

Myeloid cell lines demonstrate a stable expression of galectin-8 mRNA with no evident changes during differentiation into neutrophil-, eosinophil-, and monocyte-like cells in a model of HL-60 cells [23,26]. In the case of thrombopoietin-induced differentiation of human megakaryoblast DAMI cell line into mature megakaryocytic cells, a loss of surface bound galectin-8 was reported versus its accumulation inside the cells. This special pattern in redistribution of galectin-8 is similar to platelets which prevents internalization of platelet coagulation factor V, which is an important aspect of thrombosis and hemostasis [53].

## 8. Galectin-9

Galectin-9 is a tissue-enhanced protein as per the HPA classification, which is expressed in most tissues and cell types (Supplementary Figure S1G) and detected in human serum at basal levels varying in the range of 1–5 ng/mL [93,176]. Galectin-9 has been observed to play a diverse role in the differentiation of several cell types (Table 1 and Table 2). In macrophage differentiation, it is upregulated, suggesting that it is involved in promoting differentiation of macrophages and induced regulatory T cells. Similarly, it may be involved in promoting osteoblast differentiation. In contrast, it has an inhibitory role in the differentiation of helper T cells, osteoclasts and potentially in the myeloid cell differentiation into eosinophil lineage.

### 8.1. Macrophages

Galectin-9 levels were significantly up-regulated at the mRNA and protein level in different models of macrophage differentiation including primary human monocytes and the THP-1 human monocytic cell line [50]. This upregulation was readily observed in monocytic cells exposed to various differentiation stimuli such as natural growth factors GM-CSF and M-CSF, chemical inducer PMA, and co-culture with hepatitis C virus-infected hepatocyte cells. CD14^+^ monocyte cells treated with recombinant galectin-9 (2 µg/mL) displayed an increase in the expression of macrophage marker CD206 and in the production of M2 cytokines [93]. Galectin-9-induced M2 macrophage phenotype was also confirmed in the case of THP-1 cells. These findings have an interesting implication for cancer biology as the M2 phenotype favors tumor progression [177]. Indeed, considering that the serum level of galectin-9 was increased by an average of 3.6-fold in patients with metastatic melanoma, galectin-9 was proposed as a potential target for anticancer therapy [93].

It should be noted that in a model of human promyelocytic HL-60 cells, the levels of galectin-9 at mRNA and protein levels were significantly decreased along the eosinophilic and monocytic differentiation induced by sodium butyrate and PMA, respectively [23]. At the same time, neutrophilic differentiation of HL-60 cells induced by high dose of DMSO did not change the expression of galectin-9 [26]. The reasons for this discrepancy are not clear and need further investigations.

### 8.2. T Lymphocytes

Galectin-9 also plays a promoting role in the differentiation of induced regulatory T (iTreg) cells while suppressing differentiation of T_H_17 cells [67,96,97]. The basal levels of galectin-9 mRNA and protein expression were found to be higher in iTreg cells than in other T cell subsets. Moreover, the use of galectin-9-deficient mice and recombinant galectins revealed that the clustering of CD44 and TGF-βR1 amplified TGF-β signaling pathway and upregulation of transcription factor Foxp3, which was central in mechanisms of galectin-9-mediated differentiation of iTreg cells. Galectin-9 also might prevent iTreg from developing into effector T cells. However, galectin-9 gene knockdown cells failed to suppress T_H_1 cell response [67]. 

Galectin-9 plays a suppressive role in T_H_17 and potentially T_H_1 cell differentiation. Galectin-9 induced apoptosis of Tim-3 (T-cell immunoglobulin- and mucin-domain-containing molecule 3) expressing T_H_1 and T_H_17 cells and decreased the number of T_H_17 cells in vivo [67,96,97,178]. Galectin-9 also suppressed IL-17 mRNA expression in T_H_17 cells, signifying that T_H_17 cell differentiation from naive T cells was suppressed [97]. Similarly, T_H_1/T_H_17 differentiation and proliferation of CD4^+^ T cells was also suppressed by galectin-9 released from umbilical cord-derived mesenchymal stem cells [179]. STAT and JNK signaling pathways were vital for galectin-9 expression in mesenchymal stem cells, and were activated by IFN-γ [179]. As to T_H_2 cell differentiation, this process was promoted by co-culturing activated rat hepatic stellate cells with CD4^+^ T lymphocytes and resulted in a decrease of galectin-9 protein expression in hepatocytes after 24 h [180].

### 8.3. Osteoblasts and Osteoclasts

Recombinant galectin-9 at a concentration range of 1–100 nM induced in a dose-dependent manner osteoblast differentiation through CD44/Smad signaling pathway, which was similar to the effect of BMP-2 [94]. This signaling leads to phosphorylation of Smad1/5/8, translocation of Smad4 to the nucleus, and activation of the Id1 promoter, which in turn promotes osteoblastic differentiation. Galectin-9 expression is also upregulated in MSCs undergoing stress-induced osteoblast differentiation. A 1.6-fold increase in galectin-9 mRNA was reported at 3 h upon tensile stress applied to parietal mouse bones along with bone morphogenetic proteins, Wnt, epithelial growth factor signaling factors, and matricellular proteins [60]. Similarly, the expression of galectin-9 was increased in human MSCs cells during TGFβ3-induced differentiation into chondrocytes and recombinant galectin-9 at a concentration of 1–100 nM enhanced this differentiation through up-regulation of Smad2 signaling [41]. 

The role of galectin-9 in osteoclast differentiation is different from osteoblasts. In osteoclast precursor RAW-D and primary bone marrow cells, galectin-9 suppressed osteoclastogenesis through binding to a transmembrane protein Tim-3 [95]. This effect of galectin-9 was dose-dependent (concentration range of 1–100 nM) and carbohydrate-dependent (inhibition by lactose).

## 9. Galectin-10 (Charcot-Leyden Crystal Protein)

Galectin-10, also known as Charcot–Leyden crystal protein (gene symbol *CLC*), is considered a tissue-enriched galectin as per the HPA classification with evident protein expression primarily in bone marrow, spleen, lymph nodes, and blood cells (Figure S1H). This galectin is highly expressed in eosinophils where it plays a role in inflammatory conditions such as asthma [181]. Galectin-10 has mostly intracellular localization and is a strong marker of myeloid cell differentiation, including basophils, eosinophils, and neutrophils (Table 1). Human cell lines which express galectin-10 or demonstrated upregulation of galectin-10 during cellular differentiation are AML4, HMC-1, HL-60, and LAMA-84. Galectin-10 is also detected in human plasma at a relatively low concentration of 240 ng/L, which is detectable by mass spectrometry (Supplementary Table S1).

Studies with human promyelocytic cell line HL-60 revealed that galectin-10 expression significantly increased at both mRNA and protein levels in a time-dependent manner during eosinophil-like differentiation induced by sodium butyrate and neutrophil-like differentiation induced by DMSO [23,26]. The fold change in the expression of galectin-10 mRNA ranged from 5.5 to 7.5, respectively. Remarkably that in both cases the surface level of galectin-10 was readily enhanced indicating its secretion from the differentiated cells. In the context of eosinophilic differentiation, characteristic upregulation of galectin-10 mRNA and protein was observed in a myeloid cell line AML14 [182]. A high expression of galectin-10 mRNA was detected in a basophil-like cell line LAMA-84 [183] and human mast cell line HMC-1 [184], although no studies on detecting changes in galectin-10 expression during differentiation of these cells are available. A constitutive and dominant expression of galectin-10 was also reported in a subpopulation of CD24^+^ regulatory T lymphocytes and was required to maintain immunosuppressive functions of these cells, i.e., their specific phenotype of differentiated cells [185]. 

## 10. Galectin-12

Galectin-12 is classified by the HPA as a group-enriched gene (www.proteinatlas.org), which is expressed mostly in adipose and myeloid tissues [22,26]. In these tissues and cells, galectin-12 plays opposite roles stimulating differentiation of adipocytes and inhibiting neutrophilic differentiation of promyelocytes (Table 1), as reviewed in detail by Wan and co-authors [186].

### 10.1. Adipocytes, Sebocytes, and Colorectal Cells

Galectin-12 is a biomarker of adipocyte differentiation and is known to be co-localized with perilipin-1 on cytoplasmic lipid droplets [186]. The mouse 3T3-L1 preadipocyte cell line was used as a model system to study a relationship between galectin-12 and adipocytic differentiation induced by an adipogenic hormone mixture consisting of isobutylmethylxanthine, dexamethasone, and insulin [39]. This study revealed a significant induction of galectin-12 expression at both transcript and protein levels in mature adipocytes and an inhibitory effect of galectin-12 knockdown on different aspects of adipocyte differentiation. Thus, siRNA-induced downregulation of galectin-12 resulted in many fewer lipid droplets in 3T3-L1 cells treated with adipogenic hormones, reduced expression of adipogenic transcription factors (C/EBPα/β and PPARγ), defective adipogenic signaling through ERK, Akt, CREB, and IGF-1/insulin receptors [39]. The similar galectin-12-associated signaling pathways were proposed for differentiation of human sebocytes considering that both the sebaceous gland tissue and sebaceous gland SZ95 cells express same lipogenic factors (C/EBPα, SREBP-1, SCD, and resistin) as adipocytes [187]. The siRNA knockdown of galectin-12 was also reported to inhibit adipogenesis of porcine adipocytes by downregulating lipogenic genes (*PPARγ* and *aP2*) and activating lipid hydrolysis (triglyceride lipase and hormone-sensitive lipase) through the PKA-Erk1/2 signaling pathway [188]. Lastly, a positive association between cell differentiation (enterocyte lineage) and *de novo* galectin-12 expression was reported in eight human colorectal carcinoma cell lines (HCT15, KM12, LS180, TC7, CX1, HT-29, SW480, and SW707), which were treated with sodium butyrate [189]. 

### 10.2. Neutrophils

Galectin-12 is readily expressed in human white blood cells and in several promyelocytic cell lines (HL-60, KU-812, NB4 and U937) [21,24,25,30,190], which can undergo differentiation into three different myeloid lineages. Two cell lines, NB4 and HL-60, were used to study the role of galectin-12 in neutrophilic differentiation induced by either all-trans-retinoic acid (ATRA) or DMSO [22,26]. In contrast to adipocyte and enterocyte differentiation, DMSO-induced neutrophilic differentiation of HL-60 cells led to a downregulation of galectin-12 at both the mRNA and protein levels [26]. Moreover, the expression of galectin-12 gene in HL-60 cells under different treatment regimens correlated inversely with the expression of neutrophilic differentiation marker *NCF1*. The galectin-12 downregulation by doxycycline-inducible RNA interference enhanced ATRA-induced neutrophilic differentiation of NB4 cells, which was accompanied by the cell growth arrest, expected segmentation of nuclei morphology, elevation of neutrophilic differentiation markers (CD11b, p47^phox^), and oxidative burst activation [22]. Remarkably, the formation of lipid droplets was inhibited in differentiated NB4 cells in conjunction with inhibiting effector molecules of lipogenic signaling pathway (CREB1 and ERK) and lipogenic transcription factors (C/EBPα/β and PPARγ). Thus, galectin-12 serves as an inhibitor of neutrophilic differentiation through a mechanism which is different from stimulating adipocyte differentiation. A different cell-specific role of galectin-12 is also evident in macrophages, which is not required for the differentiation of bone marrow cells into macrophages but drives the polarization between M1 and M2 subsets [186,191]. 

## 11. Galectins-13, -14 and -16

Genes encoding galectin-13, -14, and -16 occupy a specific cluster on chromosome 19 and are considered tissue enriched galectins as per the HPA classification with predominant expression for all of them in the placenta as well as galectin-13 and galectin-16 also in brain tissues (www.proteinatlas.org). All three high number galectins are involved in differentiation of trophoblasts (Table 1) and are reported to induce T cell apoptosis and thereby likely immunosuppression during pregnancy. The altered function of this galectin gene cluster may lead to complications in pregnancy, such as preeclampsia.

The human choriocarcinoma cell line BeWo is often used as a model system to study mechanisms of trophoblast differentiation into multinucleated syncytiotrophoblasts, which can be induced by cAMP, 8-Br-cAMP, or forskolin [192]. Treatment of these cells with forskolin resulted in a significant increase of the expression of galectin-13 at protein and mRNA levels, which was inhibited by a protein kinase A inhibitor H-89 confirming cAMP-mediated signaling pathway [193]. As such, galectin-13 was proposed to be involved in the process of syncytialisation of trophoblasts driven by the transcription factor glial cells missing 1 (GCM1) and syncytin-1. The role of galectin-13 as a syncytiotrophoblast marker was also demonstrated in a different model of ACH-3P human trophoblasts cells that was dependent on forskolin-induced downregulation of LIN28A, an RNA-binding protein and regulator of embryonic stem cell differentiation [194]. The regulatory role of galectin-13 in fetal development was proposed to result from binding to a transcription factor HOXA1, which is one of the most rapidly induced genes in embryonic stem cell differentiation [195]. 

A comprehensive set of studies on placental galectins was performed by Than and co-authors that revealed crucial properties and functions of these genes and proteins in trophoblast differentiation and placenta development [4,196,197]. First, human genes encoding these galectins (*LGALS13*, *LGALS14*, and *LGALS16*) are localized in a specific cluster on chromosome 19, which is evolutionary conserved in primates. Second, galectins-13, -14, and -16 are strongly expressed in terminally differentiated syncytiotrophoblasts versus very low expression in cytotrophoblasts and the placental expression of galectin-13 and galectin-14 is decreased in women with preeclampsia. Third, the expression of placental chromosome 19 cluster galectin genes is regulated by several transcription factors including GCM1, ESRRG, GATA2, and TEF5. Fourth, all three high number galectins (-13, -14, -16) might play a role in immunosuppression at the maternal-fetal interface due to their ability to induce apoptosis of T lymphocytes. It should be noted that the role and regulation of galectin-16 is less clear because this gene does not always show the same pattern of changes in expression as galectin-13 and galectin-14, e.g., in response to a DNA methyltransferase inhibitor 5-azacytidin in BeWo cells, although forskolin increased the expression of all three genes [4]. In addition, there were higher peak expression levels for galectin-13 and galectin-14 than galectin-16 in differentiating primary trophoblast cells, but the opposite was reported for BeWo cells, with higher galectin-16 expression [4]. 

## 12. *O*-GlcNAc Cellular Homeostasis and Galectins

*O*-GlcNAcylation is a specific type of glycosylation of mostly intracellular proteins with a single monosaccharide GlcNAc, which is *O*-linked to relevant serine or threonine residues. This posttranslational modification of proteins is a highly dynamic process driven by two enzymes *O*-GlcNAc transferase (OGT) and *O*-GlcNAcase (OGA), competing for adding and removing *O*-GlcNAc, respectively [198,199]. *O*-GlcNAcylation of proteins is involved in molecular mechanisms controlling not only the stability and functionality of regulatory molecules and transcription factors but also their trafficking and localization in cells. The latter is especially essential for those soluble proteins, which can be localized inside and outside of cells like galectins. As extensive and excellent reviews of *O*-GlcNAcylation and its cell biological context are available [198,199,200], we will highlight only aspects related to cellular differentiation and galectins. 

Mammalian cells contribute significant resources (up to 5% of glucose metabolized through hexosamine biosynthetic pathway providing UDP-GlcNAc substrate) to maintain *O*-GlcNAc homeostasis in stem, stressed, and transformed cells. Remarkably that generally *O*-GlcNAc levels are significantly attenuated in many types of differentiated versus parental progenitor/stem cells regardless of the cell lineage (Table 3). Stem cells maintain a high level of *O*-GlcNAc and use different mechanisms employing *O*-GlcNAcylated proteins, including transcription factors such as OCT4, GATA-1, SOX2, NANOG, C-MYC, and KLF4 [27,201,202,203,204,205,206,207] and multiple signaling pathways including MEK-ERK1/2 signaling [208] to promote pluripotency and self-renewal. A paramount decrease of *O*-GlcNAc levels was reported during differentiation of embryonic stem cells and model cancer cell lines into haematopoietic cell lineage [31,148,205], neurons [201], muscles cells [28,209], keratinocytes [210], and enterocytes [31]. However, an increase of global O-GlcNAc was reported for osteogenic differentiation [143,144,211,212], adipocytic differentiation [213,214], chondrocytic differentiation [215], and corneal epithelial cells [216]. The reasons of this discrepancy are not clear and may be challenging in terms of a cell lineage-dependent context and the basal levels of *O*-GlcNAcylation. Indeed, the excessive *O*-GlcNAcylation attenuated BMP2-induced osteoblastic differentiation of C2C12 cells, although early stages of this process are associated with a global increase of *O*-GlcNAcylated proteins in several cellular models of osteogenesis [144,211].

**Figure 1:**
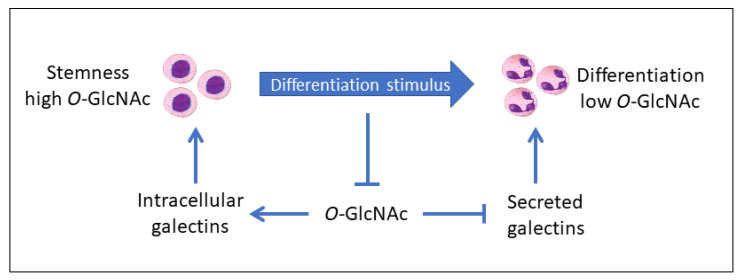
Model of *O*-GlcNAc/galectin-mediated regulation of cell stemness and differentiation. Regulatory functions of galectins toward cell self-renewal and differentiation depend on the localization of galectins in cells, which is driven by *O*-GlcNAc signaling mechanisms. Given that differentiation of multiple cell lineages is associated with decreasing levels of *O*-GlcNAcylated proteins, the model proposes that high levels of *O*-GlcNAc promote intracellular accumulation of galectins in stem and progenitor cells while low levels of *O*-GlcNAc promote galectin secretion and cell differentiation.

Conventional *O*-GlcNAcylation targets intracellular proteins in the cytoplasm, nucleus, and mitochondria that contributes to stabilization, changes the functional activities, and governs localization and trafficking of regulatory proteins and transcription factors in cells [217,218,219,220]. Some of these proteins can be secreted, however a role of *O*-GlcNAcylation remains obscure and contradictory. For instance, elevated levels of *O*-GlcNAc mitigated the secretion of insulin from rat pancreatic islets [221,222,223] whereas augmented the release of IL-8 from human mast cells [224] and the shedding of hyaluronan synthases 3 from human melanoma MV3 cells [225]. Generally, these differences might not be unexpected due to a well-known diversity of secretory mechanisms as well as properties of secreted proteins, including an option of posttranslational modification with *O*-GlcNAc. As we have described in previous sections, galectins are available in circulation and secreted from cells exposed to different differentiation stimuli. Elevated secretion or cell surface accumulation of galectins was reported during differentiation of plasma cells [66], macrophages [48], granulocytes [33], muscle cells [56,57], and adipocytes [37]. Remarkably that in the case of immune cells, the inverse relationship was mostly observed between secretion of galectins and the global levels of *O*-GlcNAc in differentiating myeloid cells. Galectins do not have an N-terminal signal sequence or hydrophobic domain for targeting the ER and therefore are secreted by unconventional transport pathways including direct non-vesical translocation across the plasma membrane via recruitment by phosphatidylinositol-4,5-bisphosphate and release in extracellular vesicles (microvesicles, exosomes, and secretory lysosomes) [226,227]. Moreover, extracellular galectins can be rapidly endocytosed and re-enter the cell [2,228,229] that may contribute to maintaining the balance between extra- and intracellular galectins. Molecular mechanisms of these events are intensively studied, however an integrated conceptual understanding is still missing and limited to a few galectin family members (galectins-1, -3, and -9). As the cell secretome is sensitive to global *O*-GlcNAc levels [230,231], we propose that the secretion of galectins can be controlled through *O*-GlcNAc-mediated mechanisms facilitating the galectin release from mature immune cells with low *O*-GlcNAc versus progenitor cells with high *O*-GlcNAc (Figure 1). Indeed, *O*-GlcNAcylation occurs mostly with intracellular proteins and prevents their aggregation [232], while cytoplasmic galectins are thought to oligomerize and accumulate beneath the plasma membrane for secretion [101,227,233]. In silico analysis using OGTSite and YinOYang 1.2 web servers, predicts that all human galectins contain serine or/and threonine residues, which can be potentially *O*-GlcNAcylated (Table 4). Although these bioinformatics tools use different algorithms for prediction of *O*-GlcNAcylation sites in proteins including support vector machine at OGTSite [234] and artificial neuronal network at YinOYang [235], there are many sites which are recognized by both programs. So far, the first experimental evidence of *O*-GlcNAcylation was provided only for galectin-1 [124] and further proof-of-principle studies to validate the reliability of computational *O*-GlcNAc predictions are required. It is important that galectins also show different gene expression profiles in low versus high *O*-GlcNAc intracellular microenvironments representing differentiated versus progenitor cells, respectively [31]. Moreover, pharmacological agents reducing *O*-GlcNAc in cells such as PMA and Ca^2+^ ionophore A23187 were shown to induce secretion of galectin-3 from THP-2 cells [236] and BHK cells [237], respectively. The functioning of *O*-GlcNAc/galectin signaling pathways in the context of cellular differentiation may be cell lineage-specific because of variations in the galectin gene and protein expression profiles and basal levels of *O*-GlcNAc in progenitor cells. For instance, a hallmark of cancer cells and stressed cells is a high level of *O*-GlcNAc, however these cells usually secrete elevated levels of galectins which can be detected in circulation or cell culture media. It is plausible that the secretion mechanism might be essential in this context as cancer cells can release multiple and aberrant *O*-GlcNAcylated proteins [238]. Cell-specific homeostatic regulation of *O*-GlcNAc signaling, *O*-GlcNAc-sensitive transcription factors, and galectin secretion mechanisms might be balanced to control cell differentiation pathways or to maintain self-renewal and pluripotency of stem cells.

## 13. Conclusions

Galectins are involved in regulating cell differentiation and maintaining cell stemness and self-renewal, however there is still a limited amount of understanding of the underlying molecular mechanisms and signaling pathways responsible for expression, redistribution, and localization of galectins in cells. Galectin gene and protein expression profiles are different between cell types and tissues and the galectin network undergo significant changes in differentiating cells, producing different phenotypes. Although the expression of galectin genes is not always drastically changed in cells similar to specific biomarkers of differentiation, posttranslational mechanisms that are involved in regulation of localization and trafficking of galectin proteins deserve emerging consideration due to often opposite or different function of galectins inside and outside the cell. Extracellular galectins can bind to the cell surface glycoreceptors exposing *N*-acetyllactosamine epitopes and regulate cellular responses via autocrine mechanisms activating transmembrane signaling [45,239,240,241,242]. This mechanism can be controlled by sialyltransferases, which add sialic acid to terminal galactose residues of N-linked glycans and thereby mask the galectin-binding sites on the cell surface and prevent galectin-mediated signaling [243,244,245]. The sialyltransferase ST6Gal-I, expression of which is enriched in stem-like cells [246], has a proven role in this regulation. Galectins secreted from the cells can also traffic back to the nucleus and exert their influence on cell development program as demonstrated for galectin-1 in mammary gland morphogenesis [245]. Inside cells, depending on *O*-GlcNAc environment, galectins can directly participate in the assembly of special components of intracellular machinery essential for regulating cell proliferation and differentiation. For instance, galectin-3 controls the cell division by stabilization of pericentrosomal matrix and spindle pole cohesion through an association with a key mitotic regulatory protein NuMA in an *O*-GlcNAc-dependent manner [247].

Molecular mechanisms that drive trafficking of galectins in cells and galectin secretion remain elusive including mechanisms of unconventional secretion of galectins that lack an ER-signal peptide. Several signaling pathways have been proposed to orchestrate galectin translocation in cells, which rely on calcium influx, protein kinase C activity, stress-activated caspase-1 among other systems [227]. These pathways may not explain all aspects of galectin distribution between intra- and extracellular spaces that is balanced by multiple cellular processes such as exocytosis, endocytosis, and non-vesicular transport [2,226,227,228,229]. We propose a new model of galectin regulation that considers the consistent change in *O*-GlcNAc homeostasis between progenitor/stem cells and mature differentiated cells. *O*-GlcNAc-mediated regulation of galectins may operate at different levels including the (a) modulation of activity of transcription factors required for galectin gene expression, (b) regulation of intracellular localization, trafficking, and secretion of galectins, (c) stability of galectin molecules, and (d) functional activity and availability of galectins for glycan-dependent and glycan-independent interactions with relevant partners. This novel approach will most likely help to integrate the current knowledge about galectin biology as an essential but underestimated stream in biomedical and life sciences due to the multifunctional properties of at least twelve human galectin members. In fact, the focus on galectins as regulatory factors of cellular differentiation promotes a potential application of galectin and galectin inhibitors for the prevention and targeted treatment of proliferative diseases, tissue regeneration, and stem-cell therapy. 

## Figures and Tables

**Table 1:** Changes in the expression of galectins associated with cellular differentiation.

Cell Lineage	Galectin	mRNA	Intracellular Galectin Levels	Extracellular Galectin Levels	References
Adipocytic differentiation of mouse 3T3-L1 preadipocyte cells induced by adipogenic medium	galectin-1 galectin-3 galectin-12	ND ↑ ↑	ND ↑ ↑	↑ ND ND	[37] [38] [39]
Adipocytic differentiation of mouse 3T3-L1 preadipocyte cells induced by linoleate	galectin-3	ND	↑	↑	[40]
Chondrocytic differentiation of human MSC induced by TGFβ3	galectin-9	ND	↑	ND	[41]
Endothelial progenitor cell differentiation of CD34^+^ mononuclear cells from the bone marrow and cord blood	galectin-3	↑	ND	ND	[42,43]
Enterocytic differentiation of HT-29 cells induced by cell crowding stress	galectin-1 galectin-2 galectin-3 galectin-4 galectin-7 galectin-8 galectin-9	↔ ↔ ↑ ↑ ↔ ↑ ↔	ND ND ND ND ND ND ND	ND ND ND ND ND ND ND	[31]
Enterocytic differentiation of human LS174T cells induced by inhibiting ribosome protein L29	galectin-4	ND	↑	ND	[44]
Eosinophilic differentiation of human HL-60 cells induced by sodium butyrate	galectin-1 galectin-3 galectin-8 galectin-9 galectin-10	↔ ↔ ↔ ↓ ↑	ND ND ND ↓ ↑	ND ND ND ↓ ↑	[23]
Erythroid differentiation of human K562 cells induced by erythropoietin or aphidicolin	galectin-1	ND	↓	↑	[45]
Granulocytic differentiation of human U937, U937T, NB4 cells induced by hypoxia	galectin-1	ND	↑	ND	[46]
Keratinocytic differentiation of primary epithelial cells at cell culture confluence	galectin-1 galectin-3 galectin-7	↓ ↑ ↑	ND ↔ ND	ND ND ND	[47]
Macrophagic (M1) differentiation of human monocytes induced by GM-CSF, IFN-γ and LPS	galectin-1 galectin-3	↔ ↔	↔ ↑	↔ ↑	[48]
Macrophagic (M1/M2) differentiation of human THP-1 cells induced by cancer cell condition media	galectin-3 galectin-9	↑ ↔	ND ND	ND ND	[49]
Macrophagic (M1/M2) differentiation of human THP-1 cells induced by PMA and primary CD4+ monocytes induced by GM-CSF and M-CSF	galectin-9	↑	ND	ND	[50]
Macrophagic (M2a/M2c) differentiation of human monocytes induced by M-CSF, IL-4 and IL-10	galectin-1 galectin-3	↔ ↑	↔ ↑	↑ ↑	[48]
Macrophagic differentiation of human THP-1 cells induced by PMA	galectin-3	↑	↑	ND	[51,52]
Megakaryocytic differentiation of human DAMI cells induced by thrombopoietin	galectin-8	ND	↔	↓	[53]
Monocytic differentiation of human HL-60 cells induced by PMA	galectin-1 galectin-3 galectin-8 galectin-9	↔ ↑ ↔ ↓	ND ↑ ND ↓	ND ↑ ND ↓	[23] [23,54] [23] [23]
Myeloid differentiation of human CD34^+^ mononuclear cells from the bone marrow	galectin-3	ND	↑	↑	[55]
Myogenic differentiation of mouse myoblast C2C12 cells induced by horse serum	galectin-1	ND	↓	↑	[56,57]
Neutrophilic differentiation of human HL-60 cells induced by DMSO	galectin-1 galectin-3 galectin-8 galectin-9 galectin-10 galectin-12	↔ ↑ ↔ ↔ ↑ ↓	↑ ↑ ND ND ↑ ↓	ND ND ND ND ND ND	[26]
Oligodendrocytic differentiation of mouse N20.1 cells induced be the elevated temperature of 39 °C	galectin-1 galectin-3	ND ND	↔ ↔	ND ND	[58]
Oligodendrocytic differentiation of rat oligodendroglial progenitor cells induced by growth factor withdrawal	galectin-1 galectin-3 galectin-4	ND ND ↓	↔ ↑ ↓	ND ND ↑	[58] [58] [59]
Osteoblastic differentiation of mouse MSC cells induced by tensile stress	galectin-9	↑	ND	ND	[60]
Osteoblastic differentiation of primary human osteoblasts cocultured with HUVECs cells	galectin-3	↓	↓	ND	[61]
Osteoblastic differentiation of rat calvaria cells induced by ascorbic acid and β-glycerophosphate	galectin-3	↑	ND	ND	[62]
Osteoclastic differentiation of mouse bone marrow cells induced by M-CSF and RANKL and human blood monocytes induced by M-CSF and RANKL	galectin-3	↑	ND	ND	[63]
Plasma cell differentiation of human BL36 cells induced by 5-azacytidine	galectin-1	ND	↑	↑	[64]
Plasma cell differentiation of mouse splenic B cells induced by LPS	galectin-1 galectin-3 galectin-8	↑ ↓ ↓	↑ ↓ ↑	↑ ND ↑	[65,66] [66] [66]
T lymphocyte (iTreg) differentiation of CD4^+^ T cells induced by TGFβ1	galectin-9	↑	↑	ND	[67]
Trophoblastic differentiation of human BeWo cells induced by forskolin	galectin-13 galectin-14 galectin-16	↑ ↑ ↑	ND ND ND	ND ND ND	[4]
Trophoblastic differentiation of mouse trophoblast stem cells induced by depleted medium	galectin-1	↑	↑	ND	[68,69]
Trophoblastic differentiation of rat Rcho-1 cells induced by horse serum	galectin-1 galectin-3 galectin-4 galectin-8 galectin-9	↔ ↔ ↓ ↔ ↔	ND ND ND ND ND	ND ND ND ND ND	[70]

Notes: ↓—upregulation, ↑—downregulation, ↔—no changes, ND—no data.

**Table 2:** Effects of recombinant galectins on cellular differentiation.

Galectin and Cell Lineages	Effect of Galectin (Concentration, Time)	Differentiation Markers	References
**Galectin-1**			
Astrocytic differentiation of immature astrocyte culture	Stimulation (10 µM, 24 h)	↑ GFAP, BDNF	[71]
Chondrogenic differentiation of human MSCs	Stimulation (0.4–2.0 µg/mL, 14 days)	↑ leucine incorporation (protein synthesis)	[72]
Chondrogenic differentiation of human MSCs	Inhibition (2.0–10.0 µg/mL, 14 days)	↓ sulfate incorporation (proteoglycan synthesis)	[72]
Fibrocytic differentiation of human monocytes	Stimulation (0.1–10 µg/mL, 5 days)	Cell morphology	[73]
Granulocytic differentiation of human and mouse bone marrow cells	Inhibition (10 μg/mL, 7–14 days)	↓ CFU-GM, BFU-E	[74]
Granulocytic differentiation of human and mouse bone marrow cells	Stimulation (1, 10, and 100 ng/mL, 7–14 days)	↑ CFU-GM, BFU-E	[74]
Granulocytic differentiation of U937T cells	Stimulation (10 µg/mL, 4 days)	↑ *GM-CSFR*, *G-CSFR*, *M-CSFR* ↑ CD11^+^ cells	[46]
Myogenic differentiation of bovine fetal MSCs	Stimulation (100 nM, 4–12 days)	↓ *MYF5* ↑ *MYOD*, *MYOG*	[75]
Myogenic differentiation of human fetal MSCs	Stimulation (200 ng/mL, 3–12 days)	↑ desmin, Myf5, Pax7, MyoD, myogenin	[24]
Myogenic differentiation of mouse primary myoblasts and C2C12 cells	Stimulation (9–10 µg/mL, 3–7 days)	↑ Multinucleated myotubes	[76,77]
Oligodendrocytic differentiation of oligodendroglial progenitor cells	Inhibition (5 µg/mL and 10 µg/mL, 48–72 h)	↓ MBP	[58]
Oligodendrocytic differentiation of oligodendroglial progenitor cells	Stimulation (125 µg/mL, 48 h)	↑ NG2, MBP, Iba-1 ↔ GFAP	[78]
Osteoblastic differentiation of bone marrow stromal cells	Stimulation (10 and 1000 ng/mL, 96 h)	↑ ALP ↓ osteocalcin	[79]
T lymphocyte (T_H_17) differentiation of CD4^+^ T cells	Inhibition (10 μg/mL, 10 days)	↓ IFN-γ and IL-17 producing cells, *RORC2*	[80]
Tolerogenic dendritic cell differentiation of human peripheral CD14^+^ monocytes	Stimulation (1 μg/mL, 5 days)	↑ IL-10, IL-6, G-CSF ↓ IL-8	[81]
Trophoblastic differentiation of BeWo cells	Stimulation (10, 30, and 60 μg/mL, 24 h)	↓ β-catenin, Ki67	[82]
Trophoblastic lineages differentiation of mouse TSC cells	Stimulation (150 ng/mL, 1–7 days)	↓ *Esrrb*, *Eomes* ↑ *Plf, Ctsq, Tpbpa*	[68]
**Galectin-2**			
Macrophage polarization to M1 phenotype from human monocytes	Stimulation (10 µg/mL, 24 h)	↑ TNF-α, IL-6, IL-12p40, INF-β, CD40	[83]
Macrophage polarization to M2 phenotype from human monocytes	Inhibition (10 µg/mL, 24 h)	↓ PDGF-C, CCL26, CD206	[83]
**Galectin-3**			
Dendritic cell differentiation of human monocytes	Inhibition (60 μg/mL, 2 days)	↓ CD1b^+^ cells ↔ HLA-DR^+^ cells	[84]
Fibrocytic differentiation of human monocytes	Stimulation (0.1–10 µg/mL, 5 days)	Cell morphology	[73]
Oligodendrocytic differentiation of oligodendroglial progenitor cells	Stimulation (5 and 10 μg/mL, 48–72 h)	↑ MBP	[58]
Osteoblastic differentiation of human fetal osteoblast cells	Inhibition (1.6 µM, 1 week)	↓ ALP, *APLP*, *RUNX2*, *SP7*, *COL1A1*, *IBSP*, *GBLAP*	[85]
Osteoclastic differentiation of mouse bone marrow cells	Inhibition (1, 2, and 5 μg/mL, 8–10 days)	Cell morphology	[63]
Osteoclastic differentiation of mouse RAW-D cells and rat bone marrow cells	Inhibition (1, 5 and 10 μg/mL, 3–4 days)	↓ NFATc1 ↓ TRAP^+^ multi-nucleated cells	[86]
Vascular smooth muscle cell transdifferentiation of pulmonary artery endothelium cell	Stimulation (5 µM, 7 days)	↑ α-SMA, calponin	[87]
**Galectin-4**			
Macrophagic differentiation of human monocytes	Stimulation (10 µg/mL, 24–48 h)	↑ CD14, CD64, CD11b, active MMP-2, TNF-α, IL-6, IL-10	[88]
Oligodendrocytic differentiation of immature oligodendrocytes	Inhibition (0.25 μM, 4 days)	Multiple markers of oligodendrocytes	[59]
**Galectin-7**			
T lymphocyte (T_H_1) differentiation of CD4^+^ T cells	Stimulation (1, 5, and 10 μg/mL, 5 days)	↓ Smad3 (nuclear), *IL-10* ↑ Smad3 (cytoplasmic), *IFN-γ*, *TNF-α*	[89]
**Galectin-8**			
Osteoblastic differentiation of naïve mouse osteoblasts	Inhibition (50 nM, 24 h–14 days)	↓ *OSX*, *OCN*, *DMP-1* ↓ ALP, RUNX2	[90]
Osteoclastic differentiation of mouse bone marrow cells	Stimulation (50 nM, 4/24 h–2/4/6 days)	↑ RANKL ↓ OPG	[91]
Plasma cell differentiation of splenic B cells	Stimulation (0.5 and 1 µM, 3 days)	↑ CD138 ↓ B220	[66]
T lymphocyte (T_H_2) differentiation of CD4^+^ T cells	Stimulation (0.1–1.5 µM, 4 days)	↑ IL-4	[92]
T lymphocyte (T_H_17) differentiation of CD4^+^ T cells	Inhibition (0.1–1.5 µM, 4 days)	↓ IL-17A	[92]
T lymphocyte (Treg) differentiation of CD4^+^ T cells	Stimulation (0.1–1.5 µM, 4 days)	↑ Foxp3	[92]
**Galectin-9**			
Chondrocytic differentiation of human MSCs	Stimulation (1, 10 and 100 nM, 14 days)	↑ *ACAN*, *COL2A1*, *COL10A1*, *SOX9*	[41]
Macrophagic (M2) differentiation of CD14^+^ monocytes and THP-1 cells	Stimulation (2 µg/m, 48–72 h)	↑ CD206, MCP-1,IL-10	[93]
Osteoblastic differentiation of human osteoblasts	Stimulation (1, 10 and 100 nM, 7 days)	↑ ALP	[94]
Osteoclastic differentiation of RAW-D cells, and bone marrow cells of rat and mouse	Inhibition (10 and 100 nM, 3–4 days)	↓ TRAP^+^ multi-nucleated cells	[95]
T lymphocyte (iTreg) differentiation of naïve CD4^+^ T cells	Stimulation (5, 20, 80 nM, 2–24 h)	↑ Foxp3^+^ cells	[67]
T lymphocyte (T_H_17) differentiation of CD4^+^IL-17^+^ T Cells	Inhibition (5 mg/kg/d, 7 days)	↓ IL-17, CCR6, IL-23R ↓ *IL-23*	[96]
T lymphocyte (T_H_17) differentiation of naïve CD4^+^CD62L^+^ T cells	Inhibition (30 nM, 4 days)	↓ *IL-17*	[97]
T lymphocyte (Treg) differentiation of naïve CD4^+^CD62L^+^ T cells	Stimulation (30 nM, 4 days)	↑ *Foxp3*	[97]

Notes: ↓—upregulation, ↑—downregulation.

**Table 3:** Changes of *O*-GlcNAc homeostasis during cellular differentiation.

Cell Lineage	*O*-GlcNAc Changes	References
Adipocytic differentiation of mouse 3T3-L1 preadipocytes	upregulation	[213,214]
Cardiomyocytic differentiation of mouse ESCs	downregulation	[209]
Chondrocytic differentiation of mouse pre-chondrogenic ATDC5 cells	upregulation	[215]
Enterocytic differentiation of human HT-29 cells	downregulation	[31]
Erythroid differentiation of G1E-ER4 cells	downregulation	[205]
Human corneal epithelial cell differentiation into mature, terminally differentiated stratified cells	upregulation	[216]
Keratinocytic differentiation of human epidermal cells	downregulation	[210]
Neural differentiation of mouse ESC	downregulation	[201]
Neural differentiation of human ESC	downregulation	[30]
Neutrophilic differentiation of human NB4, OCI-AML3, and HL-60 cells	downregulation	[31,148,205]
Osteoblastic differentiation of mouse preosteoblastic MC3T3-E1 cells	upregulation	[143,144,212]
Osteogenic differentiation of mouse C2C12 cells	downregulation	[211]
Primitive endoderm cell differentiation of mouse ESCs	downregulation	[208]
Skeletal myogenic differentiation of mouse C2C12 myoblasts	downregulation	[28]

**Table 4:** Predicted sites for *O*-GlcNAcylation in human galectins.

Galectin	NCBI Reference Sequence	OGTSite	YinOYang
Galectin-1	NP_002296.1	**S84**	**S84**
Galectin-2	NP_006489.1	S23, S51, S80, T85, S122	no predictions
Galectin-3	NP_002297.2	**S84**, T133	**S84**, S91, S92, T98, T104, T243
Galectin-4	NP_006140.1	S40	S58, T217, T317
Galectin-7	NP_002298.1	**S9**	S2, S8, **S9**, S45, T57, T58
Galectin-8	NP_006490.3	T22, S152, **T160**, T201, T211	**T160**, S178, S358
Galectin-9	NP_033665.1	**S18**, T32, **T152**, **S165**, **T193**, S202	S4, S6, S12, **S18**, S139, **T152**, S160, T161, **S165**, **T193**, T195, T351, T355
Galectin-10	NP_001819.2	**T9**	**T9**, T16
Galectin-12	NP_001136007.2	T81, **T82**, S143, **S192**, S221, **T232**, S305	**T82**, **S192**, **T232**, S315
Galectin-13	NP_037400.1	**S13**, **S119**, **S127**, **T133**	S2, S3, **S13**, **S119**, **S127**, **T133**
Galectin-14	NP_064514.1	**T9**, **S13**	S2, S3, **T9**, **S13**, S138
Galectin-16	NP_001177370.2	**S13**, **S119**	**S13**, **S119**

Notes: The bioinformatics analysis was performed using the OGTSite (http://csb.cse.yzu.edu.tw/OGTSite/) and YinOYang (http://www.cbs.dtu.dk/services/YinOYang/) online tools, which use different algorithms (support vector machine versus artificial neuronal network, respectively) for prediction of *O*-GlcNAc sites in proteins. The predicted sites present specific serine (S) or threonine (T) residues, which tentatively can be *O*-GlcNAcylated. The sites which are recognized by both tools are highlighted in bold. The specificity level of searching at OGTSite was set up as ‘high’ (90%) and a default threshold was used at YinOYang server.

## Supplementary Materials

The following are available online at https://www.mdpi.com/2073-4409/9/8/1792/s1, Figure S1: Galectin protein expression patterns in normal human tissues, Table S1: Galectins detected in the human plasma by mass spectrometry.
