# Supplementary material for: The Emerging Role of Galectins and O-GlcNAc Homeostasis in Processes of Cellular Differentiation
Source: Cells. 2020 Jul 28;9(8):1792. doi: 10.3390/cells9081792 (PMC7465113; doi:10.3390/cells9081792)
Supplement: Supplementary file 1 [file cells-09-01792-s001.pdf]

The emerging role of galectins and *O*-GlcNAc homeostasis in processes of cellular differentiation

Rada Tazhitdinova and Alexander V. Timoshenko

Department of Biology, The University of Western Ontario, London, Ontario, Canada, N6A 5B7

Table S1. Galectins detected in the human plasma by mass spectrometry

Galectin name	Concentration
Galectin-1	19 μg/L
Galectin-2	n.d.
Galectin-3	2.3 μg/L
Galectin-4	92 ng/L
Galectin-7	3.7 μg/L
Galectin-8	n.d.
Galectin-9	n.d.
Galectin-10	240 ng/L
Galectin-12	n.d.
Galectin-13	n.d.
Galectin-14	n.d.
Galectin-16	n.d.

Notes: The data were extracted (June 28, 2020) from The Human Protein Atlas portal (https://www.proteinatlas.org/humanproteome/blood/proteins+detected+in+ms); n.d., no data.

A

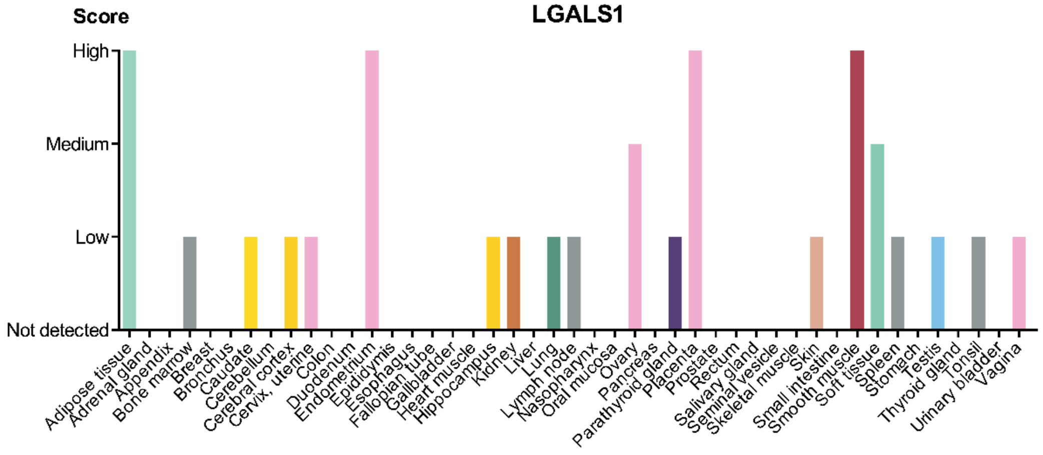
LGALS1
Score
High
Medium
Low
Not detected
Adipose tissue
Adrenal gland
Appendix
Bone marrow
Breast
Bronchus
Caudate
Cerebellum
Cerebral cortex
Cervix, uterine
Colon
Duodenum
Endometrium
Epididymis
Esophagus
Fallopian tube
Gallbladder
Heart muscle
Hippocampus
Kidney
Liver
Lung
Lymph node
Nasopharynx
Oral mucosa
Ovary
Pancreas
Parathyroid gland
Placenta
Prostate
Rectum
Salivary gland
Seminal vesicle
Skeletal muscle
Skin
Small intestine
Smooth muscle
Soft tissue
Spleen
Stomach
Testis
Thyroid gland
Tonsil
Urinary bladder
Vagina

B

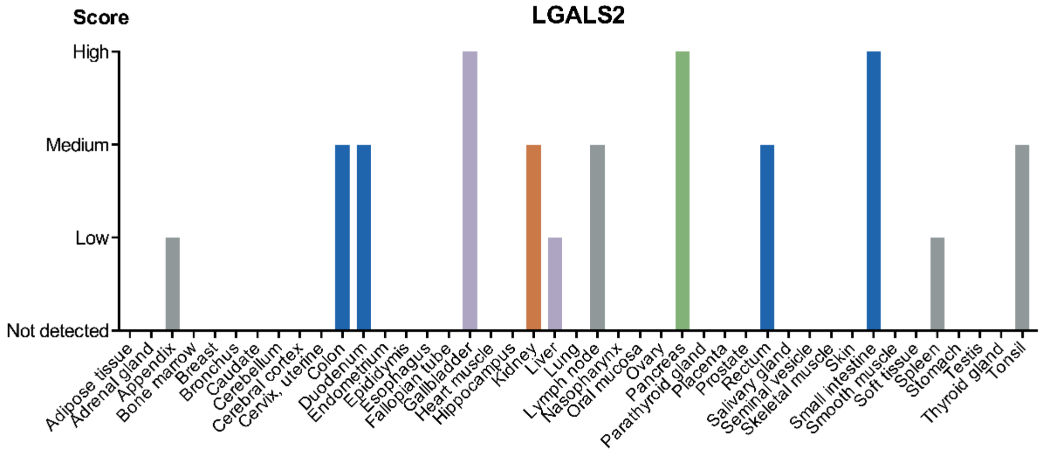
LGALS2
Score
High
Medium
Low
Not detected
Adipose tissue
Adrenal gland
Appendix
Bone marrow
Breast
Bronchus
Caudate
Cerebellum
Cerebral cortex
Cervix, uterine
Colon
Duodenum
Endometrium
Epididymis
Esophagus
Fallopian tube
Gallbladder
Heart muscle
Hippocampus
Kidney
Liver
Lung
Lymph node
Nasopharynx
Oral mucosa
Ovary
Pancreas
Parathyroid gland
Placenta
Prostate
Rectum
Salivary gland
Seminal vesicle
Skeletal muscle
Skin
Small intestine
Smooth muscle
Soft tissue
Spleen
Stomach
Testis
Thyroid gland
Tonsil

C

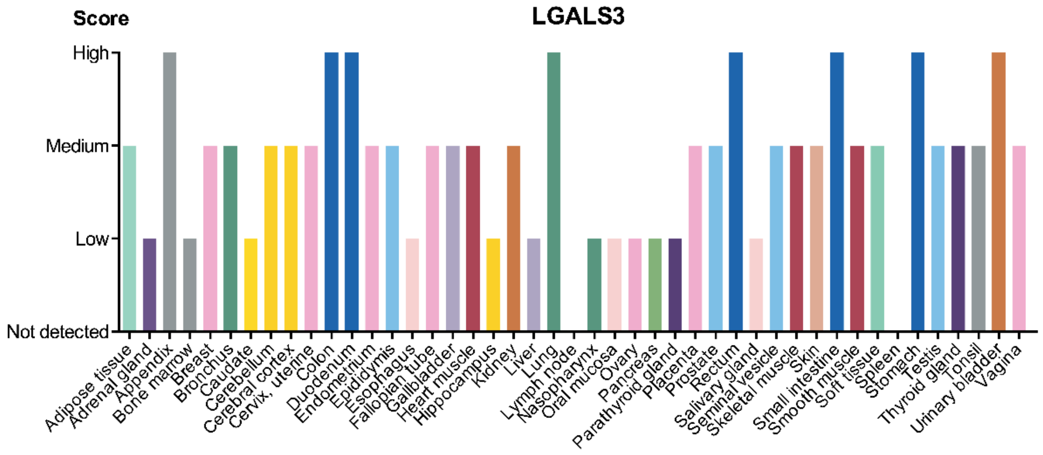
LGALS3
Score
High
Medium
Low
Not detected
Adipose tissue
Adrenal gland
Appendix
Bone marrow
Breast
Bronchus
Caudate
Cerebellum
Cerebral cortex
Cervix, uterine
Colon
Duodenum
Endometrium
Epididymis
Esophagus
Fallopian tube
Gallbladder
Heart muscle
Hippocampus
Kidney
Liver
Lung
Lymph node
Nasopharynx
Oral mucosa
Ovary
Pancreas
Parathyroid gland
Placenta
Prostate
Rectum
Salivary gland
Seminal vesicle
Skeletal muscle
Skin
Small intestine
Smooth muscle
Soft tissue
Spleen
Stomach
Testis
Thyroid gland
Tonsil
Urinary bladder
Vagina

D

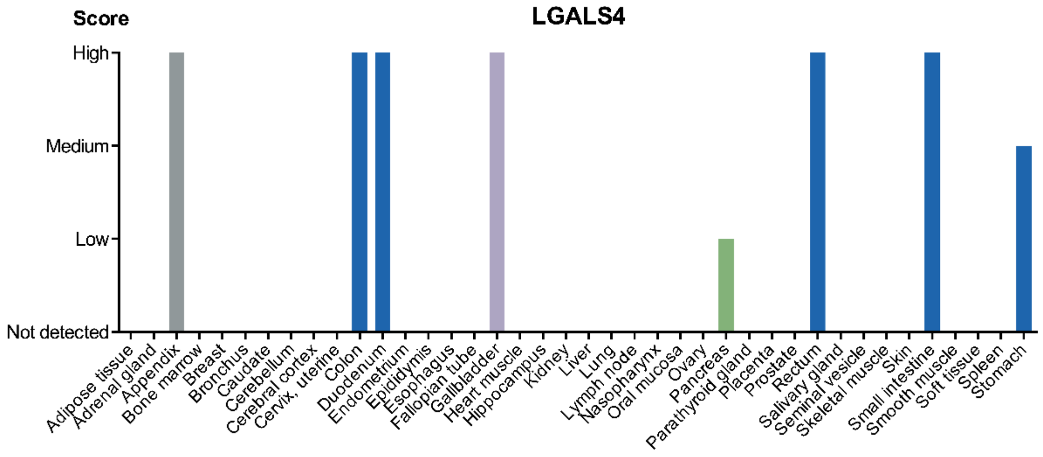
Score
LGALS4
High
Medium
Low
Not detected
Adipose tissue
Adrenal gland
Appendix
Bone marrow
Breast
Bronchus
Caudate
Cerebellum
Cerebral cortex
Cervix, uterine
Colon
Duodenum
Endometrium
Epididymis
Esophagus
Fallopian tube
Gallbladder
Heart muscle
Hippocampus
Kidney
Liver
Lung
Lymph node
Nasopharynx
Oral mucosa
Ovary
Pancreas
Parathyroid gland
Placenta
Prostate
Rectum
Salivary gland
Seminal vesicle
Skeletal muscle
Skin
Small intestine
Smooth muscle
Soft tissue
Spleen
Stomach

E

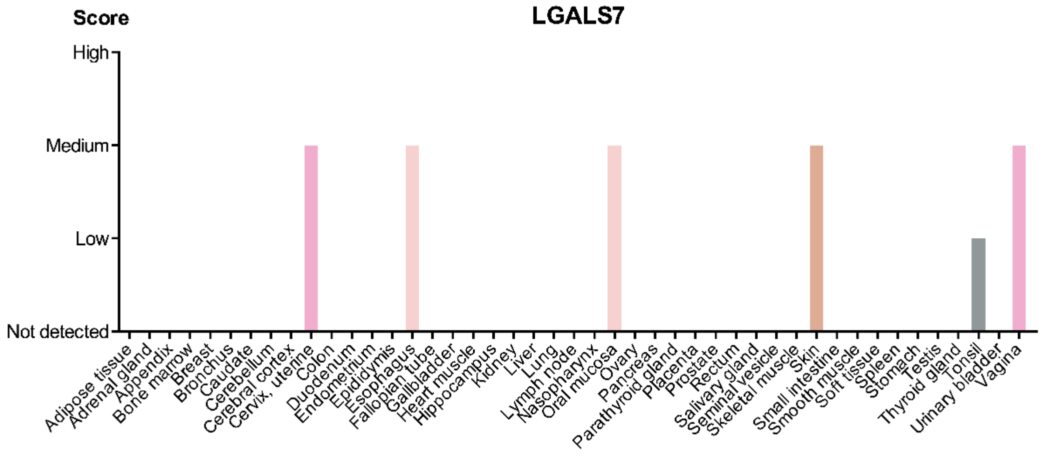
Score
LGALS7
High
Medium
Low
Not detected
Adipose tissue
Adrenal gland
Appendix
Bone marrow
Breast
Bronchus
Caudate
Cerebellum
Cerebral cortex
Cervix, uterine
Colon
Duodenum
Endometrium
Epididymis
Esophagus
Fallopian tube
Gallbladder
Heart muscle
Hippocampus
Kidney
Liver
Lung
Lymph node
Nasopharynx
Oral mucosa
Ovary
Pancreas
Parathyroid gland
Placenta
Prostate
Rectum
Salivary gland
Seminal vesicle
Skeletal muscle
Skin
Small intestine
Smooth muscle
Soft tissue
Spleen
Stomach
Testis
Thyroid gland
Tonsil
Urinary bladder
Vagina

F

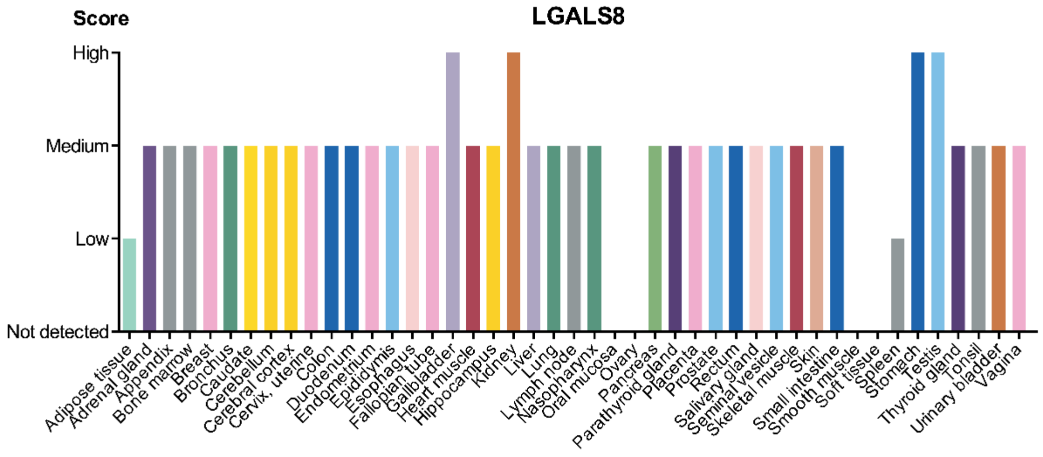
Score
LGALS8
High
Medium
Low
Not detected
Adipose tissue
Adrenal gland
Appendix
Bone marrow
Breast
Bronchus
Caudate
Cerebellum
Cerebral cortex
Cervix, uterine
Colon
Duodenum
Endometrium
Epididymis
Esophagus
Fallopian tube
Gallbladder
Heart muscle
Hippocampus
Kidney
Liver
Lung
Lymph node
Nasopharynx
Oral mucosa
Ovary
Pancreas
Parathyroid gland
Placenta
Prostate
Rectum
Salivary gland
Seminal vesicle
Skeletal muscle
Skin
Small intestine
Smooth muscle
Soft tissue
Spleen
Stomach
Testis
Thyroid gland
Tonsil
Urinary bladder
Vagina

G

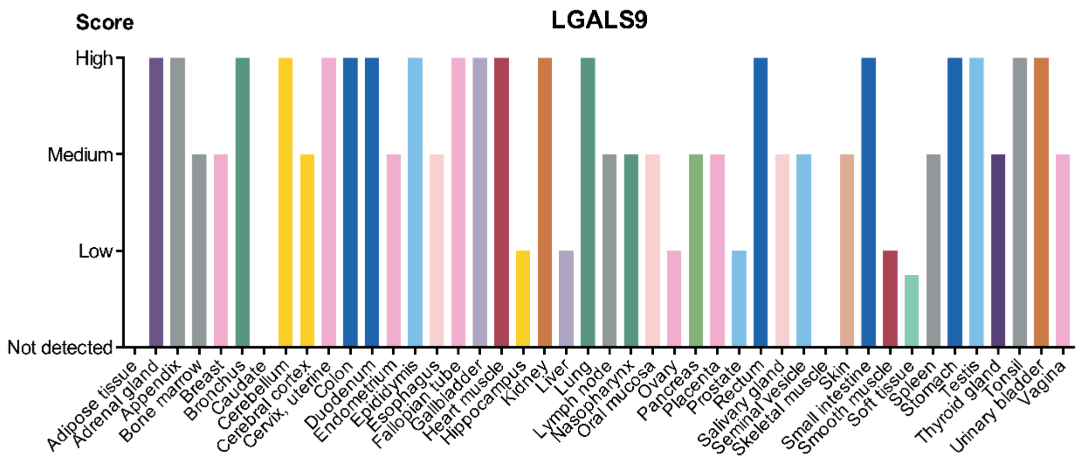

H

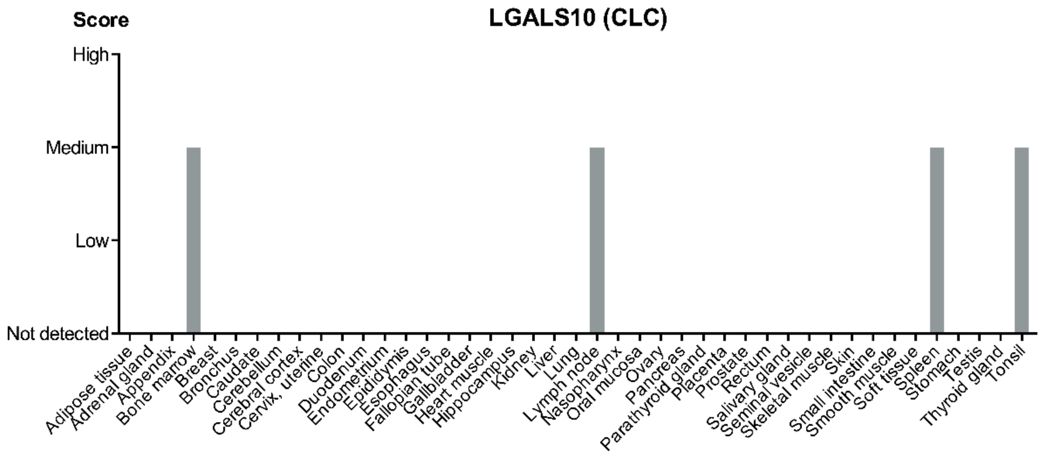

Figure S1. Galectin protein expression patterns in normal human tissues. The data were extracted from The Human Protein Atlas portal, Protein Atlas version 19.3 (https://www.proteinatlas.org/). **A**, galectin-1, low tissue specificity; **B**, galectin-2, group enriched tissue specificity; **C**, galectin-3, low tissue specificity; **D**, galectin-4, group enriched tissue specificity; **E**, galectin-7, group enriched tissue specificity; **F**, galectin-8, low tissue specificity; **G**, galectin-9, low tissue*/tissue enhanced specificity; **H**, galectin-10, tissue enhanced specificity. The Human Protein Atlas does not list data on the expression of galectins 12, 13, 14, and 16 at protein levels. The primary reference: Uhlén M et al, 2015. **Tissue-based map of the human proteome.** *Science* PubMed: 25613900 DOI: 10.1126/science.1260419
